# Integrating plan-do-check-act (pdca)—based closed-loop management into lifestyle intervention for pediatric primary obesity

**DOI:** 10.3389/fped.2026.1723732

**Published:** 2026-04-10

**Authors:** Hongxian Lv, Tingting Xu, Haiying Fang, Yiping Ni, Weibin Guo, Xiang Li

**Affiliations:** 1 Third Department of Pediatrics, Jinhua Maternal and Child Health Care Hospital, Jinhua, Zhejiang Province, China; 2 Internal Medicine Department, Tianjin Corps Hospital of the Chinese People’s Armed Police Force, Tianjin, China; 3 Department of Pediatric Rehabilitation, Jinhua Maternal and Child Health Care Hospital, Jinhua, Zhejiang Province, China; 4 Binzhou Center for Disease Control and Prevention, Binzhou, Shandong Province, China; 5 Guozhong Kangjian (Liaoning) Health Technology Co., Ltd., Shenyang, China

**Keywords:** childhood obesity, lifestyle intervention, metabolic indicators, PDCA cycle, weight management

## Abstract

**Background:**

Childhood primary obesity is a major public health issue. Traditional lifestyle interventions often fail due to lack of systematic and continuous individualization. The Plan-Do-Check-Act (PDCA) cycle, a continuous quality improvement tool, may offer a new approach for its long-term management.

**Objective:**

This study aimed to retrospectively compare the effectiveness of the PDCA closed-loop management model for childhood primary obesity.

**Method:**

This study retrospectively analyzed the clinical data of 100 children with primary obesity World Health Organization (WHO) underwent PDCA closed-loop management in the pediatric healthcare department of Jinhua maternal and child health care hospital from January 2022 to December 2024. The children's information was retrospectively collected and divided into the PDCA closed-loop management group (*n* = 50) and the Non-PDCA routine management group (*n* = 50). The observation period was 6 months. Anthropometric indicators: [weight, height (calculating body mass index z score, BMI-Z score)], body fat percentage (measured by bioelectrical impedance method), waist circumference; biochemical indicators: fasting blood glucose (FBG), fasting insulin (FINS, calculating homeostatic model assessment of insulin resistance, HOMA-IR), lipid profile [total cholesterol (TC), triglycerides (TG), low-density lipoprotein cholesterol (LDL-C), high-density lipoprotein cholesterol (HDL-C)]; behavioral assessment: using questionnaires to evaluate dietary behavior and screen time. The differences in each indicator before and after the implementation of this management method were compared.

**Result:**

Compared with before treatment, all indicators improved significantly after treatment (*P* < 0.05): BMI-Z score, body fat percentage, and waist circumference all decreased significantly (*P* < 0.05). Metabolic indicators improved significantly, including LDL-C, TG, HOMA-IR, and triglyceride levels decreased, and HDL-C levels increased (*P* < 0.05). The questionnaire survey showed that the daily activity intensity significantly increased, and the frequency of unhealthy snack intake and daily screen time significantly decreased (*P* < 0.05).

**Conclusion:**

The PDCA closed-loop management model, through continuous monitoring, evaluation, and individualized adjustment, can significantly improve the body composition, metabolic parameters, and related behavioral habits of obese children. Integrating this into the lifestyle of children with primary obesity to achieve long-term weight management is feasible and effective.

## 1 Introduction

Childhood obesity is a common chronic disease, referring to a state where, during childhood, energy intake exceeds energy expenditure for an extended period, resulting in excessive accumulation of fat in the body and causing substantial harm to health. According to the definition of the World Health Organization (WHO), this disease has been clearly classified as a complex pathological condition, rather than a simple physical characteristic (1). In clinical practice, the body mass index (BMI) is commonly used as an important basis for screening and diagnosis. The specific diagnostic criteria are as follows: A child is defined as having overweight if his or her BMI falls within the 85th to less than the 95th percentile for age and sex. Obesity is diagnosed when the age- and sex-specific BMI is at or above the 95th percentile, according to Centers for Disease Control and Prevention (CDC) growth charts (2). This standard was formulated based on large-scale epidemiological survey data and has a clear evidence-based medical basis. The etiological mechanism of this disease is complex and diverse, mainly involving the interaction of multiple factors. Genetic factors constitute the basic susceptibility for the occurrence of the disease. Studies have shown that children whose parents (either both or one) are obese have a significantly increased risk of developing the disease (3). Environmental and behavioral factors are the main driving forces behind the occurrence of diseases, including but not limited to excessive intake of high-energy-density diets, increased consumption of sugary beverages, insufficient intake of dietary fiber, and significant reduction in physical activity (4). Furthermore, social and economic development factors also exert an indirect influence by affecting food accessibility and lifestyle choices. Childhood primary obesity has become one of the most serious public health challenges globally in the 21st century. Its wide prevalence, rapid growth rate, and profound impact on individual health and the social economy have far exceeded the scope of traditional aesthetics, and have been clearly defined by the World Health Organization as a complex chronic disease (5). In recent years, with significant changes in lifestyle and dietary patterns, the incidence of childhood obesity has been continuously rising, showing a trend of younger age and wider prevalence (6). Global data shows that over the past 40-plus years, the obesity rate among children and adolescents has increased by more than 8 times, and the growth has been particularly rapid in low-income and middle-income countries (7). As a rapidly developing major country with a strong economy, China has witnessed a rapid transformation in children's nutritional status, moving from “malnutrition” to “overnutrition”. The “Report on Nutrition and Chronic Diseases of Chinese Residents” and related studies indicate that the rate of overweight and obesity among children and adolescents aged 6–17 in China has approached 20%, and in some developed urban areas, it even exceeds 30%, showing a significant upward trend, which foretells a huge future disease burden and public health pressure (8, 9).

The hazards of childhood obesity are multifaceted, systemic, and have far-reaching consequences. Physiologically, obese children often exhibit pathological changes such as insulin resistance, abnormal glucose tolerance, lipid disorders, non-alcoholic fatty liver disease, hypertension, and early atherosclerosis. These traditional “adult diseases” have a significantly increased incidence rate among children, seriously compromising their quality of life and long-term health conditions (10). Obesity can also affect the endocrine system of children, leading to problems such as precocious puberty and abnormal growth and development (11). On the psychological and social level, obese children are more likely to be ridiculed, discriminated against, and even subjected to school bullying due to their body shape. This leads to psychological issues such as low self-esteem, anxiety, and depression. Social avoidance and behavioral problems are also more common. These have long-term negative impacts on their personality development, academic performance, and social adaptability (12). In the long term, childhood obesity exhibits a clear “trajectory phenomenon”, with approximately 70%–80% of obese children continuing to be obese into adulthood, significantly increasing their risk of suffering from serious chronic diseases such as cardiovascular and cerebrovascular diseases, diabetes, and certain cancers. This not only shortens their expected lifespan but also imposes a heavy economic burden on families and the social healthcare system (13).

At present, both domestic and international guidelines unanimously emphasize that lifestyle-based treatment is the preferred and fundamental treatment option for children with primary obesity (14). Developed countries started their intervention efforts for childhood obesity earlier and have developed many systematic intervention models. For instance, family-based behavioral intervention has been proven to be an effective strategy. It emphasizes parental involvement, goal setting, self-monitoring, and the application of incentive mechanisms (15). Furthermore, comprehensive intervention programs at the community and school levels, which aim to create a healthy environment through multi-sectoral collaboration, have also achieved certain results (16). In terms of management tools, foreign scholars have been actively exploring the use of mobile health technologies, such as smartphone applications and wearable devices, for remote monitoring and behavioral prompts, to enhance the convenience and compliance of interventions (17). However, these measures also face challenges, such as high project costs, difficulties in maintaining long-term effects, and significant differences in outcomes among families with different socioeconomic backgrounds (17). More importantly, many of these measures still exhibit “fragmented” characteristics, lacking a continuous improvement framework that can integrate assessment, planning, implementation, feedback, and adjustment, making it difficult to achieve the optimal treatment effect (18).

Research and practice on the treatment of childhood obesity in China are also in the process of exploration. Common models include hospital outpatient guidance, school health education, and community health promotion activities, etc. (19). In recent years, Chinese scholars have also gradually recognized the significance of family involvement and behavior correction, and have carried out a series of treatment studies centered on families. At the same time, drawing on experience, some treatment models combining internet technology have also emerged, such as conducting health education and follow-up management through the WeChat platform (20, 21). However, in general, there are still several shortcomings in China's treatment practices: The treatment mainly focuses on knowledge dissemination and short-term centralized management, lacking long-term, systematic tracking and dynamic adjustment mechanisms, resulting in a widespread “separation of knowledge and action” phenomenon (22). The management model is mostly led by medical staff. The active participation and self-management ability of the children and their families have not been fully stimulated, and compliance is difficult to maintain (22). The evaluation of treatment efficacy is mostly limited to anthropometric indicators such as weight, height, and BMI. There is insufficient comprehensive assessment of body composition, metabolic indicators, and behavioral changes, making it difficult to fully reflect the actual effectiveness of the treatment (14). These limitations mean that the long-term effectiveness and cost-effectiveness of treating childhood obesity in our country still need to be improved.

In this context, the “plan-do-check-act” cycle from the field of quality management has demonstrated its great potential and adaptability as a framework for managing lifestyle treatments for childhood obesity. The Plan-Do-Check-Act (PDCA) cycle consists of a scientific, logical, and continuously improving closed-loop management system. The model's fundamental principle is an iterative four-stage cycle. These stages are closely connected, with each repetition building on previous outcomes to achieve continuous quality improvement (23, 24). This mature management model can be creatively applied to the health management of childhood obesity, and its internal logic is highly compatible: in the planning stage, based on a comprehensive assessment, both the doctor and the patient jointly identify the core issues, analyze the root causes, and formulate specific, measurable, achievable, relevant, and time-bound individualized goals and action plans; in the implementation stage, the family acts as the main body to implement the plan, and medical staff provide remote support and light supervision through modern communication technologies to ensure the continuity of the implementation; in the inspection stage, systematic and regular multi-dimensional data are collected, and objective data is used to accurately assess the stage results, and the gap between the execution and the plan goals is strictly examined; in the handling stage, successful experiences are standardized and solidified, the existing problems are analyzed for their root causes, and based on this, the treatment strategies for the next cycle are adjusted and optimized (25–27). The PDCA cycle, through its inherent closed-loop nature, continuity, and spiral upward trend, precisely compensates for the shortcomings of traditional treatment models. It transforms one-off behavioral prescriptions into a “continuous quality improvement” project with goals, execution, feedback, and correction, extending medical treatment from a static “point” to a dynamic, self-optimizing “cyclic system”, providing an extremely practical theoretical framework and practical path for achieving long-term and effective management of childhood obesity (27, 28).

Given the increasingly serious problem of childhood obesity and the limited effectiveness of traditional interventions, this study innovatively applied the PDCA closed-loop management model to the lifestyle intervention for children with primary obesity. The aim was to systematically evaluate the actual effectiveness, feasibility, and advantages of this model through a retrospective case analysis. The main objectives of the study included: assessing the improvement degree of anthropometric indicators (such as BMI, waist circumference, etc.) in obese children using the PDCA model; exploring its positive impact on metabolic health conditions (such as blood sugar, blood lipids); analyzing the promoting effect of this model on obesity-related behaviors such as healthy diet and regular exercise; and summarizing the application experience and challenges of PDCA in clinical practice, providing a basis for subsequent promotion.

The innovation of this study lies in three aspects: first, the integration of theoretical tools across different fields, for the first time introducing the classic PDCA quality management model systematically into the treatment scenarios of Chinese children with obesity, achieving the integration of management methods and clinical practice; second, it constructed an operational dynamic closed-loop management model, forming a continuous cycle of “evaluation—planning—implementation—monitoring—feedback—adjustment”, breaking through the limitations of traditional static interventions in long-term compliance; third, it established a multi-dimensional comprehensive evaluation system, comprehensively evaluating the intervention effect from multiple perspectives such as anthropometry, body composition, metabolic indicators, and behavioral changes, avoiding the limitations of relying solely on weight indicators.

In the current era, when childhood obesity has become a global public health challenge, this study aims to explore the PDCA structured and dynamic management model to provide new ideas and local empirical evidence for optimizing clinical practice, thereby contributing to improving children's long-term health and reducing the burden of social diseases. The systematic application of quality management methods in the health field not only helps to enhance the efficacy of childhood obesity intervention but also provides a reference model for the management of other chronic diseases and has important theoretical and practical value.

## 2 Research subjects and methods

### 2.1 Research subjects

**Figure 1:**
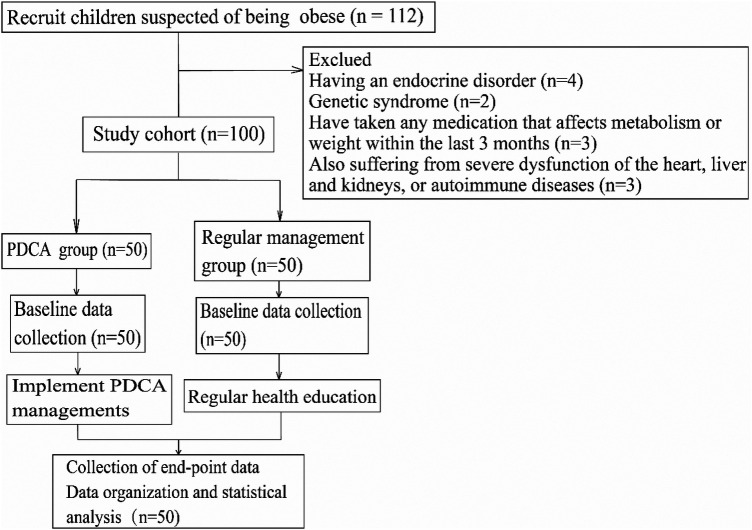
Flowchart the research process began by screening 100 children with primary obesity through standard inclusion and exclusion criteria and completing the baseline assessment. Subsequently, these patients were naturally divided into the PDCA group and the control group. The PDCA group received a 6-month closed-loop intervention, implementing the complete cycle of “plan—execute—check—act” every month, with the plan dynamically adjusted based on the assessment results; the control group received the same period of routine health guidance and follow-up. After the intervention, the anthropometric, biochemical, and behavioral indicators of both groups were systematically collected for comparative analysis.

This study is a single-center retrospective cohort analysis. It systematically collected and analyzed the data of children with primary obesity admitted to the pediatric health care department of Jinhua maternal and child health care hospital from January 2022 to December 2024. According to the different management models they received, they were divided into two groups: one group received closed-loop management integrated with the PDCA cycle (PDCA group), and the other group received conventional health education management (conventional management group) (Figure 1).

### 2.2 Inclusion and exclusion criteria

Inclusion criteria (29): (1) age range between 6 and 14 years old; (2) diagnosed as having obesity according to the “Classification Standard for Body Mass Index Values for Overweight and Obesity in Chinese School-Age Children and Adolescents” formulated by the China Obesity Problem Working Group; (3) the obesity is of a simple nature and excludes various pathological obesity; (4) good compliance with management, able to complete at least 6 months of follow-up and possess complete follow-up records; (5) the guardian gives informed consent and voluntarily participates in this study.

Exclusion criteria (1, 30, 31) (1) having endocrine disorders (such as Cushing's syndrome, hypothyroidism), (2) having genetic syndromes (such as Prader-Willi syndrome), (3) having chromosomal abnormalities or other pathological conditions that may cause obesity; (4) having severe dysfunction of the heart, liver, or kidneys or autoimmune diseases simultaneously; (5) having taken drugs that affect metabolism or weight within the recent 3 months, such as glucocorticoids, antipsychotic drugs, etc.; (6) having severely lacking clinical data and being unable to conduct effective analysis.

### 2.3 Research methodology

In this retrospective study, the subjects were divided into two groups based on their previous medical records, and each group received different health management strategies. Among them, the PDCA group received a systematic, closed-loop management treatment based on the plan-execution-check-act cycle. The specific implementation steps and precautions are as follows.

During the planning stage, the first step was to form a multidisciplinary management team. This team consisted of pediatric attending physicians, clinical nutritionists, and nursing health managers. They conducted in-depth communication with the child and his/her primary caregiver during the first visit. Based on the results of the first comprehensive assessment (including detailed physical measurements, body composition analysis, biochemical test reports, and behavioral baseline questionnaire surveys), the team discussed with the family and formulated a highly individualized treatment plan. This plan clearly set specific quantitative goals for the initial treatment period, such as the range of total daily calorie intake (calculated based on the measured basal metabolic rate and appropriately reduced), the proportion of macronutrients (suggested that carbohydrates provide 50%–55% of calories, proteins account for 15%–20%, and fats account for 25%–30%), the cumulative daily moderate-to-high-intensity physical activity time (not less than 60 min), and specific behavioral change goals (such as limiting recreational screen time to within 2 h per day). All the set goals followed the principles of being specific, measurable, achievable, relevant, and time-bound, and were recorded in the individual's health management file.

After entering the execution phase, uniform health management diaries were distributed to the families of the patients, and detailed instructions were given on how to record. Patients were required to accurately record the types of all food consumed, including estimated quantities and meal times, the types, duration, and subjective intensity of physical activities, as well as daily screen time for entertainment. To strengthen the support and supervision during the execution, the research team established a dedicated WeChat management group. The team's nutritionist or health management specialist would take turns to be on duty every day in the group, check the summary of the diaries uploaded by the families, promptly answer any questions encountered during the execution process, and provide positive and encouraging feedback. A piece of graphic and textual health knowledge popularization information made by the team would be pushed to the group every week. It is worth noting that during the execution process, the team particularly emphasized that the “truthfulness” of the records was more important than “perfection”, and encouraged the families to record even if they did not reach the standards, so as to accurately analyze the difficulties in the future.

The inspection stage was systematically integrated into the follow-up plan. The patients were scheduled to visit the fixed clinic at the fixed time every month for re-evaluation. Each re-evaluation was conducted by the same trained measurement personnel using the same calibrated set of equipment to complete all the physical measurements (height, weight, waist circumference, hip circumference) and body composition analysis (using the same InBody 770 bioelectrical impedance analyzer). At the same time, the health management specialist guided the patients or their guardians to fill out a short behavioral questionnaire again. In addition, follow-up venous blood samples were collected from fasting patients at 3 and 6 months post-treatment as scheduled, for reassessment of biochemical metabolic indicators including FBG, insulin, and lipid profile. All the test data were promptly and accurately entered into their personal health records for comparison with the target values of the previous cycle.

The processing stage is a crucial link that connects the previous stage with the next one. After collecting data during each follow-up visit, the management team will promptly hold a brief case discussion meeting. The current examination results will be compared and analyzed with the goals set in the planning stage. For projects that have met the standards or shown significant progress, the team will summarize their successful experiences (such as a family adopting “healthy snack boxes” instead of traditional snacks), provide positive reinforcement through verbal praise or small rewards, and encourage them to solidify such good behaviors into family habits. For projects that have not met the standards, the team will, in combination with the health diary records, review and analyze the reasons together with the family (such as: was the goal setting too aggressive? Was there a break in execution due to holiday gatherings? Were there any insurmountable execution obstacles?). Based on this analysis results, the team will make targeted adjustments to the treatment plan for the next cycle (for example: for children entering the weight loss plateau phase, add twice-weekly resistance training to the exercise plan; for children whose diet control was disrupted during the holiday, jointly rehearse and formulate strategies for dietary choices in social situations). Thus, a new and optimized PDCA cycle will be initiated.

During the entire treatment period, several key aspects to be noted include: Firstly, ensure smooth communication within the multidisciplinary team, maintain high consistency in management suggestions for the children, and avoid transmitting contradictory information to the families. Secondly, emphasize the establishment of a partnership-based trust relationship with the families, highlighting that the role of the management team is “supporter” and “coach” rather than “judger”, in order to alleviate the psychological pressure of the children and their parents and improve long-term compliance. Finally, all personalized plan adjustments, communication guidance contents, and reason analyses are required to be recorded in detail in the medical records, which provides valuable and comprehensive data sources for this retrospective study.

The conventional management group received standardized treatment: at the first visit, the attending physician provided about 20–30 min of education on obesity-related health knowledge and handed out printed general dietary and exercise advice brochures. They were instructed to return to the hospital for re-examination every month. During the re-examination, the outpatient nurse or physician measured height and weight, and gave simple verbal encouragement (such as “Continue to adhere to controlling your diet and doing more exercise”), without conducting systematic data monitoring, structured feedback, and dynamic adjustment of individualized plans. All the treatment and follow-up contents of both groups were recorded in the hospital information system.

### 2.4 Detection indicators

According to the medical records used for the retrospective analysis in this study, the detection indicators, measurement procedures, and clinical judgment criteria for the two groups of research subjects are as follows. All tests were conducted in the corresponding departments following the established operational procedures of the hospital. The data were collected by trained medical staff and recorded in the unified standardized medical records.

Anthropometric indicators are the basic assessment items. Height measurement requires the child to remove shoes and hats, stand upright, with the back against a vertical height gauge, with the heels, buttocks, and shoulder blades touching the gauge column, the head placed at the eye and ear plane, and the headboard moved gently to read the number, accurate to 0.1 centimeter. Weight measurement uses a calibrated electronic scale, instructing the child to remove coats and heavy clothing, stand steadily in the center of the scale pan, and read the value after it stabilizes, accurate to 0.1 kilograms. Based on the height and weight measurement values, the BMI (kg/m^2^) is calculated. To further standardize the assessment, the Body Mass Index Z (BMI-Z) Score (i.e., the standard deviation score of BMI relative to the healthy reference population of the same age and gender) is calculated according to the age and gender recorded in the medical records, referring to the “Chinese Classificaton Criteria for Overweight and Obesity Screening of School-Age Children and Adolescents”. This score is the core indicator for judging the degree of obesity and its dynamic changes. The measurement of waist circumference and hip circumference uses an inelastic tape measure. The waist circumference is measured at the midpoint level of the line connecting the upper edge of the iliac crest and the lower edge of the twelfth rib, with the reading taken at the end of exhalation; the hip circumference is measured at the level of the most protruding part of the buttocks. During measurement, the tape measure should be tightly attached to the skin but without compression, accurate to 0.1 centimeter, and the waist-to-hip ratio is calculated accordingly.

The body composition indicators were obtained through bioelectrical impedance analysis. According to the medical records, this test was completed using the same InBody 770 body composition analyzer. The measurement requirements were as follows: it should be conducted more than 2 h after a meal, and before the test, the child was instructed to empty the bladder, avoid strenuous exercise, and remove any personal metal items. The child stood barefoot on the foot electrode of the instrument, held the hand electrodes with both hands, let the arms hang naturally and slightly outward, and remained still for about 1–2 min. The instrument automatically analyzed and printed the report, recording key parameters such as body fat percentage, body fat mass, and fat-free weight.

Biochemical metabolic indicators require the collection of fasting venous blood samples. The medical records show that before blood collection, the child was instructed to fast for 8–12 h and could only drink a small amount of water. The nurse from the laboratory collected 5 mL of blood from the elbow vein, and the blood sample was centrifuged and separated into serum according to the specified process, then tested using the Roche Cobas series of automatic biochemical immunoanalyzer. The test items and clinical significance interpretation are as follows: fasting blood glucose (FBG) is measured using the glucose oxidase method, which is used for the initial screening of abnormal glucose metabolism; fasting insulin (FINS) is measured using chemiluminescence immunoassay, and together with FBG values, it is used to calculate the homeostatic model assessment of insulin resistance [HOMA-IR = FBG (mmol/L) × FINS (μIU/mL)/22.5], and HOMA-IR > 2.5 usually indicates the presence of insulin resistance; the lipid profile includes total cholesterol (TC), triglycerides (TG), high-density lipoprotein cholesterol (HDL-C), and low-density lipoprotein cholesterol (LDL-C), all of which are determined by enzymatic methods, and their judgment is based on the reference ranges in the Chinese Expert Consensus on the Prevention and Treatment of Dyslipidemia in Children and Adolescents.

The behavioral indicators were evaluated through questionnaire surveys. The medical records contained the “Child Health Behavior Questionnaire” filled out jointly by the children and their guardians at the baseline, 3 months after treatment, and 6 months after treatment. This questionnaire was a tool designed by our hospital and had undergone reliability and validity tests in previous studies. The questionnaire mainly investigated three aspects: Firstly, the cumulative time (in minutes) spent on moderate to high-intensity physical activities (such as brisk walking, running, swimming, ball games, etc., measured by slight sweating and increased heart rate and breathing) on average each day in the past week; Secondly, the average daily entertainment screen time (in minutes) spent on watching TV, using computers, tablets, and mobile phones; Thirdly, the frequency (times per week) of consuming high-sugar and high-fat snacks (such as candies, cakes, chips, fried foods, sugary beverages, etc.). The questionnaire was distributed by health managers in the outpatient department and was retrieved after on-site verification of completeness.

The test results of all these indicators were clearly recorded in the personal electronic medical records and paper health management archives of each research subject. In this retrospective study, the researchers systematically extracted and entered the values of each indicator at the three time points (baseline, 3 months, and 6 months) into the database according to the established data extraction form for subsequent statistical analysis. The accuracy of the data was controlled through the method of double independent entry and cross verification.

### 2.5 Sample size calculation

This study is a retrospective study, and all cases that meet the criteria will be included as much as possible. According to the study by Matina Kouvari et al., the standardized mean difference (SMD) = −0.61 (32). Using the G*Power software for calculation, with *α* = 0.05 (two-tailed) and *β* = 0.20 (80% test power), the required sample size for each group is approximately 34 cases, and the total sample size is 68 cases. Considering the possible data missing and loss to follow-up in the retrospective study, this study finally included 50 cases in each group, totaling 100 sample cases, to meet the basic requirements of statistical analysis and have a certain test power.

### 2.6 Statistical methods

The statistical analysis methods will be carried out using the SPSS software. Data such as anthropometric indicators, biochemical metabolic indicators, daily screen time, and moderate-to-high-intensity physical activities, which are measurement data following a normal distribution, will be described by the mean ± standard deviation. Comparisons between groups will be conducted using the independent sample *t*-test, and comparisons within groups before and after treatment will be conducted using repeated measures analysis of variance. For measurement data that do not follow a normal distribution, they will be described by the median and interquartile range, and comparisons between groups or within groups will be conducted using the Mann–Whitney *U*-test or the Wilcoxon signed-rank test. Gender and the frequency of intake of unhealthy snacks will be described by frequency and percentage, and comparisons between groups will be conducted using the chi-square test or Fisher's exact test. All statistical analyses will be considered to have statistical significance when *P* < 0.05.

### 2.7 Ethical statement

This study has been reviewed and approved by Jinhua Maternal And Child Health Care Hospital's Ethics Review Committee [Approval Number: (2021-KY036)]. This study is a retrospective study, and all data are historical clinical records. The research process will not have any impact on the patients' treatment plans. The researchers strictly followed the ethical guidelines of the Helsinki Declaration and anonymized all research data, removing all information that could identify the patients' personal identities (including but not limited to names, ID numbers, medical record numbers, contact numbers, and addresses), and only entered and analyzed the data in a numbered form. All data access and processing were conducted in a confidential environment, and the researchers have all signed confidentiality agreements. This study has no potential conflicts of interest.

## 3 Result

### 3.1 Comparison of baseline information between the two groups of patients

Statistical analysis showed that before the start of treatment, there were no statistically significant differences in all observed indicators between the PDCA closed-loop management group and the Non-PDCA conventional management group (*P* > 0.05). In terms of demographic characteristics, the average age of the two groups was similar (approximately 9.2 years), and the gender distribution ratio was comparable (the ratio of male to female was close to 1:1), indicating that the two groups were comparable in terms of the basic growth and development stage and gender composition. Obesity degree and body composition: the key obesity indicators of the two groups, including weight, height, BMI, BMI-Z score, body fat rate, and waist circumference, had very similar means, and the statistical test confirmed no differences (*P* > 0.05). This indicates that the severity of obesity and body composition distribution of the two groups of children were matched before treatment. Metabolic health status: the core indicators reflecting glucose and lipid metabolism, such as HOMA-IR, TG, HDL, and LDL levels, also had no significant differences between the two groups (*P* > 0.05). This suggests that both groups of children were at similar metabolic risk levels before treatment. Behavioral habits: although there were slight numerical differences in daily screen time and moderate-to-high-intensity physical activity time between the two groups, there was no statistical significance. And before the start of treatment, there was no statistically significant difference in the distribution of unhealthy snack intake frequency between the two groups (*P* > 0.05). The vast majority of children had the behavior of frequently consuming unhealthy snacks. More than 90% of the children consumed unhealthy snacks three times or more per week. The intake behavior was highly concentrated: “frequent (5–6 times/week)” and “very frequent (once a day or more)” were the two main categories. In the PDCA group, 64.0% (32/50) of the children, and in the Non-PDCA group, 62.0% (31/50) of the children were in these two high-frequency intervals. Only a very small number of children (less than 6% in both groups) could consume only 1–2 times or less per week. This indicates that both groups had common adverse behavioral patterns before treatment, such as prolonged sitting, excessive screen time, and frequent intake of unhealthy snacks, as shown in Table 1. The high degree of balance of baseline data is of crucial significance. It indicates that the PDCA group and the Non-PDCA group were completely comparable at the treatment start. Any differences observed between the two groups in outcome indicators, such as the extent of BMI reduction and improvement in metabolic indicators, after the treatment will mainly be attributed to the different treatment management models applied (PDCA closed-loop management vs. conventional management), rather than due to inherent differences in the baseline conditions of the two groups of patients. This greatly enhances the internal validity and reliability of the results of this retrospective study and provides a solid premise and foundation for subsequent arguments on the superiority of PDCA closed-loop management.

**Table 1:** Comparison of baseline information between the two groups of patients.

Baseline		PDCA group (*n* = 50)	Non-PDCA group (*n* = 50)	Test	95%C		Effect size	*P*-value
					Lower	Upper		
Age		9.26 ± 1.946	9.14 ± 1.917	*T*-test	−0.647	0.887	0.311	0.757
Gender (*n*, %)	Male	27 (54.00)	26 (52.00)	Chi-squared		0.040	0.841	
	Female	23 (46.00)	24 (48.00)					
Human measurement indicators								
Weight (kg)		52.35 ± 8.675	51.15 ± 9.27	*T*-test	−2.364	4.764	0.668	0.506
Height (cm)		142.52 ± 10.34	140.84 ± 11.52	*T*-test	−2.674	6.014	0.763	0.447
BMI (kg/m^2^)		24.81 ± 2.56	24.52 ± 2.74	*T*-test	−7.625	1.342	0.547	0.586
BMI-Z score		2.45 ± 0.35	2.43 ± 0.38	*T*-test	−0.126	0.166	0.271	0.787
Body fat percentage (%)		35.66 ± 4.81	35.93 ± 5.18	*T*-test	−2.256	1.716	−0.270	0.788
Waist circumference (cm)		82.45 ± 7.84	81.84 ± 8.13	*T*-test	−2.555	3.785	0.385	0.701
Biochemical metabolic indicators								
HOMA-IR		3.81 ± 1.24	3.69 ± 1.35	*T*-test	−0.394	0.635	0.466	0.642
TG (mmol/L)		1.65 ± 0.45	1.69 ± 0.47	*T*-test	−0.224	0.145	−0.428	0.670
HDL (mmol/L)		0.98 ± 0.18	0.96 ± 0.20	*T*-test	−0.046	0.105	0.785	0.435
LDL (mmol/L)		2.85 ± 0.60	2.91 ± 0.65	*T*-test	−0.318	0.179	−0.556	0.579
Behavioral indicators								
Daily screen time (minutes)		215.48 ± 75.76	225.48 ± 79.52	*T*-test	−40.764	20.764	−0.645	0.520
Moderate-to-high intensity activities (minutes per day)		34.52 ± 19.28	30.50 ± 18.43	*T*-test	−3.464	11.504	1.066	0.289
Unhealthy dietary intake frequency	Never or very rarely	0 (0.00)	0 (0.00)	Chi-squared			0.468	0.926
	Less	3 (6.00)	2 (4.00)					
	Offen	15 (30.00)	17 (34.00)					
	Frequently	22 (44.00)	20 (40.00)					
	Very frequently	10 (20.00)	11 (22.00)					

Never or very rarely is Less than once per week. Less is 1–2 times per week. Ofen is 3 to 4 times per week. Frequently is 5–6 times per week. Very frequently is 5 to 7 times per week. TG is Triglyceride. HDL is High-density lipoprotein. LDL is Low-density lipoprotein.

### 3.2 Comparison of anthropometric indicators between the two groups of patients

**Figure 2:**
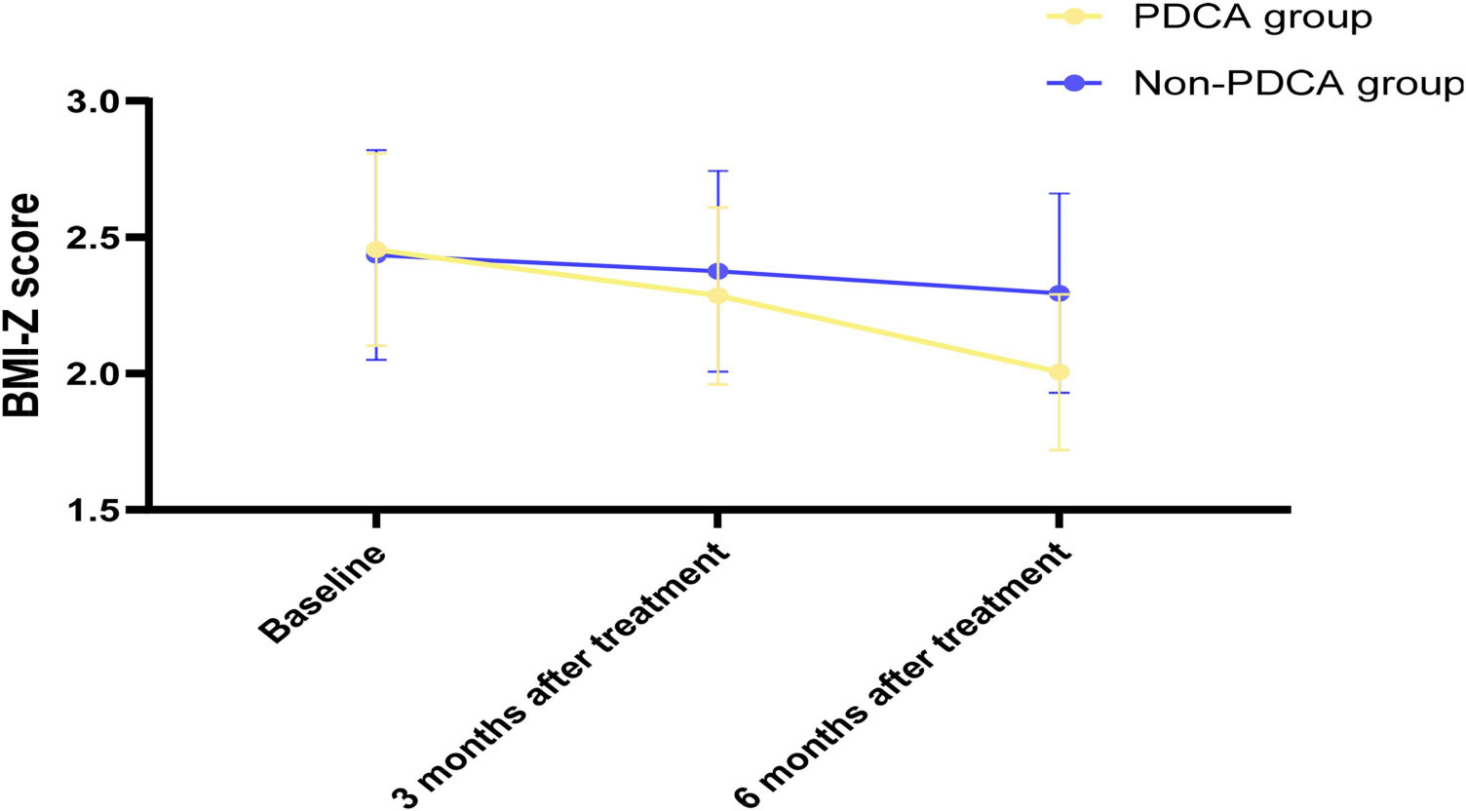
Trends in BMI-Z scores betweenc and Non-PDCA children at baseline, 3 and 6 months after treatment.

The results of the anthropometric indicators in this study showed that after 6 months of treatment, the PDCA closed-loop management group achieved significantly better outcomes in weight control, body composition optimization, and central obesity improvement compared to the conventional management group. BMI (kg/m^2^): the PDCA group gradually decreased from the baseline of 24.8 ± 2.5 to 23.9 ± 2.3 at 3 months (*P* > 0.05) and 22.5 ± 2.1 at 6 months (*P* < 0.05). The Non-PDCA group only slightly decreased from the baseline of 24.5 ± 2.7 to 23.8 ± 2.5 at 6 months (*P* > 0.05). BMI-Z score: the PDCA group significantly decreased from 2.45 ± 0.35 to 2.29 ± 0.32 at 3 months (*P* < 0.05) and 2.01 ± 0.29 at 6 months (*P* < 0.05). The Non-PDCA group only slightly decreased from 2.43 ± 0.38 to 2.30 ± 0.36 at 6 months (*P* > 0.05). Body fat percentage (%): the PDCA group decreased from 35.6 ± 4.8 to 33.2 ± 4.5 at 3 months (*P* > 0.05) and 30.1 ± 4.0 at 6 months (*P* < 0.05). The Non-PDCA group only slightly decreased from 35.9 ± 5.1 to 34.0 ± 4.7 at 6 months (*P* < 0.05). Waist circumference (cm): The PDCA group decreased from 82.5 ± 7.8 to 80.1 ± 7.2 at 3 months (*P* < 0.05) and 76.1 ± 6.5 at 6 months (*P* < 0.01). The Non-PDCA group only slightly decreased from 81.8 ± 8.1 to 79.5 ± 7.6 at 6 months (*P* < 0.05), as shown in Table 2 and Figure 2.

**Table 2:** Comparison of two groups of human measurement indicators.

Parameters		PDCA group (*n* = 50)	Non-PDCA group (*n* = 50)	95%CI		Effect size	*P*-value
				Lower	Upper		
BMI (kg/m^2^)	T_0_	24.81 ± 2.56	24.52 ± 2.74	−0.762	1.342	0.547	0.586
	T_1_	23.93 ± 2.34	24.23 ± 2.61	−1.274	0.694	−0.585	0.560
	T_2_	22.55 ± 2.13^a^^,^^b^	23.79 ± 2.54	−2.178	−0.321	−2.671	0.009
Within-group repeated measures analysis of variance	<.001	0.364					
BMI-Z score	T_0_	2.45 ± 0.35	2.43 ± 0.38	−0.126	0.166	0.271	0.787
	T_1_	2.29 ± 0.32^a^	2.38 ± 0.37	−0.228	0.048	−1.298	0.197
	T_2_	2.01 ± 0.29^a^^,^^b^	2.30 ± 0.37	−0.420	−0.145	−4.424	<.001
Within-group repeated measures analysis of variance	<.001	0.219					
Body fat percentage (%)	T_0_	35.66 ± 4.81	35.93 ± 5.18	−2.256	1.716	−0.270	0.788
	T_1_	33.21 ± 4.52	35.23 ± 4.92^a^	−3.855	−0.125	−2.117	0.037
	T_2_	30.07 ± 3.98^a^^,^^b^	34.02 ± 4.72^a^^,^^b^	−5.684	−2.215	−4.519	<.001
Within-group repeated measures analysis of variance	<.001	0.095					
Waist circumference (cm)	T_0_	82.45 ± 7.84	81.84 ± 8.13	−2.555	3.785	0.385	0.701
	T_1_	80.12 ± 7.23^a^	80.05 ± 7.62^a^	−2.880	3.020	0.047	0.065
	T_2_	76.14 ± 6.53^a^^,^^b^	79.51 ± 7.62^a^	−6.177	−0.544	−2.368	0.020
Within-group repeated measures analysis of variance	<.001	0.308					

T_0_ is the baseline period. T_1_ is three months after the intervention_._ T_2_ is six months after the intervention. BMI is body mass index. BMI-Z score is body mass index Z score. Intra-group measurements were analysed using repeated measures analysis of variance, with *P*-values reported.

^a^ Indicates that compared with the baseline of the same group, *P* < 0.05.

^b^ Indicates compared with the Non-PDCA group of the same period, *P* < 0.05.

### 3.3 Comparison of two groups of biochemical metabolic indicators

**Figure 3:**
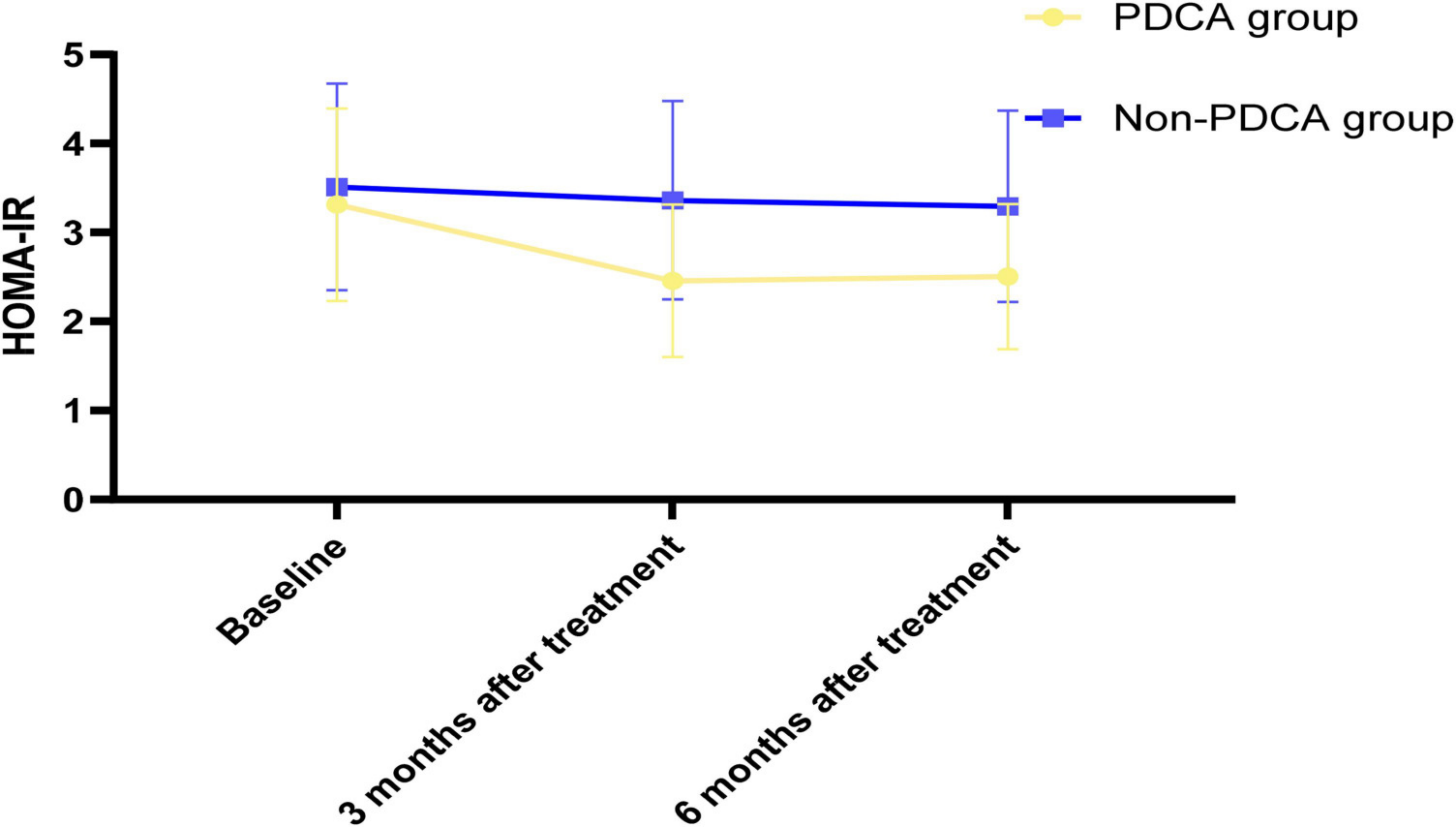
Trends in HOMA-IR scores between PDCA and Non-PDCA children at baseline, 3 and 6 months after treatment.

This study conducted dynamic monitoring of the core biochemical indicators reflecting glycolipid metabolism. The results consistently indicated that the PDCA closed-loop management could fundamentally improve the metabolic disorders related to children's primary obesity. The effect was significant and far superior to the conventional management. HOMA-IR was significantly improved: the HOMA-IR value of children in the PDCA group decreased significantly from the baseline of 3.8 ± 1.2 to 3.2 ± 1.0 at 3 months (*P* < 0.05), and further dropped to 2.5 ± 0.8 at 6 months (*P* < 0.05). In the control group, only a slight decrease from 3.7 ± 1.4 to 3.1 ± 0.9 was observed at 6 months (*P* < 0.05), and the difference between the two groups was extremely significant (*P* < 0.05). Triglycerides (TG): the TG value of the PDCA group continuously decreased from 1.65 ± 0.45 mmol/L to 1.25 ± 0.35 mmol/L at 6 months (*P* < 0.05). High-density lipoprotein cholesterol (HDL-C): the HDL-C value of the PDCA group continuously increased from 0.98 ± 0.18 mmol/L to 1.15 ± 0.16 mmol/L at 6 months (*P* < 0.05), with a significant increase. The control group only slightly increased to 1.05 ± 0.18 mmol/L (*P* < 0.05). Low-density lipoprotein cholesterol (LDL-C): the LDL-C value of the PDCA group continuously decreased from 2.85 ± 0.60 mmol/L to 2.32 ± 0.51 mmol/L at 6 months (*P* < 0.01). The control group decreased to 2.54 ± 0.59 mmol/L (*P* < 0.05), as shown in Table 3 and Figure 3. HOMA-IR is the core indicator for evaluating insulin sensitivity. The continuous and significant decrease in HOMA-IR of the PDCA group indicates a substantial improvement in insulin sensitivity. The reduction in TG and the increase in HDL-C, and the significant decrease in LDL-C. These indicate that PDCA treatment effectively regulates lipid metabolism through comprehensive lifestyle changes.

**Table 3:** Comparison of biochemical metabolic indicators between the two groups of patients.

Parameters		PDCA group (*n* = 50)	Non-PDCA group (*n* = 50)	95%CI		Effect size	*P*-value
				Lower	Upper		
HOMA-IR	T_0_	3.81 ± 1.24	3.69 ± 1.35	−0.394	0.635	0.466	0.642
	T_1_	3.22 ± 1.03^a^	3.64 ± 1.24	−0.873	0.033	−1.872	0.069
	T_2_	2.51 ± 0.82^a^^,^^b^	3.05 ± 0.85	−0.871	−0.208	−3.231	0.002
Within-group repeated measures analysis of variance	<.001	0.070					
TG (mmol/L)	T_0_	1.65 ± 0.45	1.69 ± 0.47	−0.224	0.145	−0.428	0.670
	T_1_	1.48 ± 0.38	1.53 ± 0.46	−0.219	0.120	−0.584	0.561
	T_2_	1.25 ± 0.35^a^^,^^b^	1.36 ± 0.38^a^	−0.245	0.045	−1.371	0.174
Within-group repeated measures analysis of variance	<.001	0.002					
HDL (mmol/L)	T_0_	0.98 ± 0.18	0.96 ± 0.20	−0.046	0.105	0.785	0.435
	T_1_	1.05 ± 0.17	0.98 ± 0.19	−0.002	0.142	1.928	0.057
	T_2_	1.15 ± 0.16^a^^,^^b^	1.05 ± 0.18	0.042	0.177	3.211	0.002
Within-group repeated measures analysis of variance	<.001	0.050					
LDL (mmol/L)	T_0_	2.85 ± 0.60	2.91 ± 0.65	−0.318	0.179	−0.556	0.579
	T_1_	2.58 ± 0.55	2.87 ± 0.63	−0.513	−0.047	−2.388	0.019
	T_2_	2.32 ± 0.51^a^^,^^b^	2.54 ± 0.59^a^	−0.438	−0.002	−1.999	0.048
Within-group repeated measures analysis of variance	<.001	0.006					

T_0_ is the baseline period. T_1_ is three months after the intervention_._ T_2_ is six months after the intervention. HOMA-IR is the homeostatic model assessment of insulin resistance. TG is Triglyceride. HDL is High-density lipoprotein. LDL is Low-density lipoprotein. Intra-group measurements were analysed using repeated measures analysis of variance, with *P*-values reported. Between-group comparisons were performed using independent-samples *t*-test.

^a^ Indicates that compared with the baseline of the same group, *P* < 0.05.

^b^ Indicates compared with the Non-PDCA group of the same period, *P* < 0.05.

### 3.4 Comparison of behavioral indicators between the two groups of patients

The dynamic monitoring results of three behavioral indicators—screen time, physical activity, and snack intake—in this study indicate that the PDCA closed-loop management model can efficiently and continuously promote the establishment of healthy lifestyles among children with obesity. Its behavioral correction effect is significantly better than that of conventional management. For the PDCA group, the daily screen time of children decreased significantly from the baseline of (215 ± 75) minutes to (150 ± 60) minutes at 3 months (*P* < 0.05), and further dropped to (105 ± 45) minutes at 6 months (*P* < 0.05). For the control group, it only slightly decreased from (225 ± 80) minutes to (141 ± 52) minutes at 6 months (*P* < 0.05). By the end of the treatment, the screen time of the PDCA group was significantly less than that of the control group (*P* < 0.001). For the daily moderate-to-high-intensity activity time, it increased significantly from the baseline of (35 ± 20) minutes to (50 ± 25) minutes at 3 months (*P* < 0.01), and reached (70 ± 30) minutes at 6 months (*P* < 0.001), exceeding the 60-minute daily standard recommended by the World Health Organization. For the control group, it only slightly increased from (31 ± 18) minutes to (53 ± 20) minutes at 6 months (*P* < 0.05). The difference between the groups was extremely significant at 6 months (*P* < 0.001). A significant behavioral improvement was observed by the 3-month mark. The proportion of high-risk categories (“frequent” and “very frequent”) of children decreased sharply from 64.0% to 14.0%. At the same time, the proportion of healthy categories (“less intake”) of children increased significantly from 6.0% to 36.0%, and 10.0% of the children reached the ideal state of “never or rarely” intake. This indicates that more than half of the children successfully reduced their intake frequency to less than twice a week within a short period. At 6 months: The optimization trend further strengthened and stabilized. The total proportion of children in the healthy categories (“never/very rarely” and “less”) increased from 46.0% at 3 months to 74.0%, becoming the absolute majority. The proportion of high-risk category children further decreased to 6.0%. This indicates that the vast majority of children have successfully established and maintained a new healthy diet model with low intake frequency. From the distribution perspective, at 6 months, 74.0% of the children in the PDCA group could control their intake to ≤2 times per week, while only 38.77% of the control group could do so (*P* < 0.05), as shown in Table 4.

**Table 4:** Comparison of behavioral indicators between the two groups of patients.

Parameters			PDCA group (*n* = 50)	Non-PDCA group (*n* = 50)	Test	95%CI		Effect size	*P*-value
						Lower	Upper		
Daily screen time (minutes)	T_0_		215.48 ± 75.46	225.48 ± 79.52	*T*-test	−40.764	20.764	−0.645	0.520
	T_1_		150.50 ± 59.64^a^	179.50 ± 69.55^a^	*T*-test	−54.713	−3.287	−2.238	0.027
	T_2_		105.48 ± 44.50^a^^,^^b^	141.48 ± 52.48^a^^,^^b^	*T*-test	−55.311	−16.689	−3.699	<.0.001
Within-group repeated measures analysis of variance			<.001	<.001					
Moderate-to-high intensity activities (minutes per day)	T_0_		34.52 ± 19.28	30.50 ± 18.43	*T*-test	−3.464	11.504	1.066	0.289
	T_1_		49.50 ± 23.52^a^	40.50 ± 21.50^a^	*T*-test	0.056	17.944	1.997	0.049
	T_2_		70.42 ± 29.85^a^^,^^b^	52.52 ± 20.49^a^^,^^b^	*T*-test	7.738	28.062	3.496	0.001
Within-group repeated measures analysis of variance			<.001	<.001					
Unhealthy dietary intake frequency	T_0_		0.98 ± 0.18	0.96 ± 0.20	*T*-test	−3.464	11.504	1.066	0.289
	T_1_		1.05 ± 0.17^a^	0.98 ± 0.19^a^	*T*-test	0.056	17.944	1.997	0.049
	T_2_		1.15 ± 0.16^a^^,^^b^	1.05 ± 0.18^a^^,^^b^	*T*-test	7.738	28.062	3.496	0.001
Within-group repeated measures analysis of variance			<.001	<.001					
Frequency of intake of unhealthy snacks	T_0_	Never or very rarely	0 (0.00)	0 (0.00)	Chi-squared			0.468	0.926
		Less	3 (6.00)	2 (4.00)					
		Offen	15 (30.00)	17 (34.00)					
		Frequently	22 (44.00)	20 (40.00)					
		Very frequently	10 (20.00)	11 (22.00)					
	T_1_	Never or very rarely	5 (10.00)	2 (4.08)	Chi-squared			13.324	0.010
		Less	18 (36.00)	8 (16.33)					
		Ofen	20 (40.00)	19 (38.78)					
		Frequently	7 (14.00)	17 (34.69)					
		Very frequently	0 (0.00)	4 (8.00)					
	T_2_	Never or very rarely	12 (24.00)	4 (8.16)	Chi-squared			16.750	0.002
		Less	25 (50.00)	15 (30.61)					
		Ofen	10 (20.00)	15 (30.61)					
		Frequently	3 (6.00)	13 (26.53)					
		Very frequently	0 (0.00)	3 (6.00)					
Within-group repeated measures analysis of variance			<.001	<.001					

Never or very rarely is Less than once per week. Less is 1–2 times per week. Ofen is 3 to 4 times per week. Frequently is 5–6 times per week. Very frequently is 5 to 7 times per week. T_0_ is the baseline period. T_1_ is three months after the intervention_._ T_2_ is six months after the intervention.

^a^ Indicates that compared with the baseline of the same group, *P* < 0.05.

^b^ Indicates compared with the Non-PDCA group of the same period, *P* < 0.05.

## 4 Discussion

Childhood primary obesity has become one of the most serious global public health challenges in the 21st century. Its wide prevalence, rapid growth rate, and profound impact on individual health and the social economy have far exceeded the scope of traditional aesthetics, and it has been clearly defined by the World Health Organization as a complex chronic disease. Global data showthat over the past four decades, the obesity rate among children and adolescents has increased by more than eight times, and the growth has been particularly rapid in low-income and middle-income countries (33, 34). As a rapidly developing major country with a strong economy, China has witnessed a rapid transformation in children's nutritional status, moving from “malnutrition” to “overnutrition” (35). The “Report on Nutrition and Chronic Diseases of Chinese Residents” and related studies indicate that the overweight and obesity rate among children and adolescents in China has reached approximately 20%. In some developed urban areas, it even exceeds 30%, showing a significant upward trend, which foretells a huge future disease burden and public health pressure (36, 37). The harm of childhood obesity is multi-faceted, systemic, and far-reaching. It not only causes physiological problems such as insulin resistance, abnormal glucose tolerance, and lipid disorders, but also leads to psychological and social issues such as low self-esteem, anxiety, and depression. Furthermore, approximately 70%–80% of children with obesity carry the condition into adulthood, substantially elevating their lifetime risk of developing serious chronic diseases, including cardiovascular and cerebrovascular diseases as well as diabetes (37, 38).

At present, both domestic and international guidelines unanimously emphasize that lifestyle-based treatment is the preferred and fundamental treatment option for children with primary obesity (14). Developed countries started their efforts in treating childhood obesity earlier and have established various systematic treatment models, including family-based behavioral therapy, comprehensive treatment programs at the community and school levels (39). In recent years, the application of mobile health technologies has provided new ideas for the treatment of childhood obesity. However, these treatment measures also face challenges, such as high project costs, difficulties in maintaining long-term effects, and significant differences in outcomes among families with different socioeconomic backgrounds. More importantly, many treatments still exhibit “fragmented” characteristics, lacking a continuous improvement framework that can integrate assessment, planning, implementation, feedback, and adjustment (40). There are still several shortcomings in China's treatment practices: The treatment mainly focuses on knowledge dissemination and short-term centralized management, lacking long-term, systematic tracking and dynamic adjustment mechanisms, resulting in a widespread “separation of knowledge and action” phenomenon; The management model is mostly led by medical personnel, and the active participation and self-management ability of children and their families have not been fully stimulated; The evaluation of treatment effects mainly focuses on anthropometric indicators such as weight, height, and BMI, while the comprehensive assessment of body composition, metabolic indicators, and behavioral changes is insufficient (41, 42).

This study innovatively introduced the “Plan-Do-Check-Act” cycle model from the field of quality management into the lifestyle treatment of children with primary obesity, constructing a closed-loop management system. The innovation mainly lies in three aspects: Firstly, the innovative transplantation and integration of theoretical tools, for the first time systematically and completely introducing the classic PDCA quality management cycle into the clinical outpatient treatment scenarios for Chinese children with primary obesity; Secondly, the closed-loop and dynamic construction of the management model, emphasizing the dynamic, continuous, and responsive nature of the treatment, aiming to fundamentally solve the problems of long-term compliance and effect maintenance; Finally, the systematic and comprehensive evaluation dimensions, constructing a comprehensive evaluation system covering anthropometric indicators, body composition, metabolic parameters, and behavioral changes. The significance of this study lies in providing a practical and operational treatment model for the clinical management of childhood obesity, and also offering a reference model and method for the health management of other chronic diseases.

From the perspective of anthropometric indicators, the results of this study show that the PDCA closed-loop management group achieved significantly better effects than the conventional management group in weight control, body composition optimization, and central obesity improvement. The BMI, BMI-Z score, body fat rate, and waist circumference of the children in the PDCA group showed significant decreases at 3 months of treatment and further significant improvements at 6 months. Particularly notable is the decrease in BMI-Z score, indicating that the treatment not only achieved absolute weight reduction but also shifted the weight growth curve of the children away from the original obesity trajectory and towards the healthy weight range of the same age and gender. The significant reduction in body fat rate and the significant reduction in waist circumference suggest that the PDCA treatment effectively reduced the accumulation of visceral fat, which is of great significance for reducing the risk of long-term metabolic diseases. These findings are consistent with the results of a recent systematic review by Fatemeh Kazeminasab et al., which found that combined dietary and exercise treatment can significantly reduce waist circumference (43). However, in contrast to the findings of another study by Kylie E Hunter et al., which showed that a parent-centered early childhood obesity prevention program had no significant impact on children's BMI (44), The success of the PDCA model in this study may lie in its establishment of a closed-loop system involving multiple parties (including children, families, and medical teams) with continuous feedback.

The dynamic monitoring results of biochemical metabolic indicators show that the PDCA closed-loop management can fundamentally improve the metabolic disorders related to children's primary obesity. The HOMA-IR value of children in the PDCA group significantly decreased, indicating a substantial improvement in insulin sensitivity, and blocking the key pathological physiological process leading to type 2 diabetes. At the same time, the lipid profile also changed favorably: triglyceride levels decreased, while HDL-C levels increased. This change pattern indicates that the PDCA treatment corrects the metabolic disorders related to obesity at the physiological level, significantly reducing the cardiovascular metabolic risk. These biochemical indicators' improvements are not isolated; they have a close intrinsic relationship: PDCA management first achieves energy negative balance by promoting healthy diet and increasing exercise, resulting in reduced body fat; the reduction of visceral fat directly improves the sensitivity of target organs such as the liver and muscles to insulin; the improvement of insulin sensitivity further promotes normal lipid metabolism (26, 45, 46). This “metabolic repair” effect holds crucial significance for safeguarding the long-term health of children.

The dynamic monitoring results of behavioral indicators show that the PDCA closed-loop management model can efficiently and continuously promote the establishment of healthy lifestyles among children with obesity. Children in the PDCA group significantly reduced their daily screen time, significantly increased their daily moderate-to-high-intensity activity time, and significantly decreased the frequency of unhealthy snack intake per week. There is a synergistic effect among these behavioral changes: reducing screen time increases activity opportunities, reducing snack intake reduces the burden on exercise, and together creates a huge energy negative balance. The success of the PDCA model in behavior correction is attributed to its ability to transform abstract “healthy diet” recommendations into executable, monitorable, and adjustable specific actions. Through self-monitoring via health diaries, it enhances family awareness of intake behaviors; through monthly reviews, it provides opportunities for analyzing situations and formulating coping strategies. This continuous behavioral shaping and reinforcement is the key to helping families break through the “easy to know but difficult to do” bottleneck.

Compared with previous studies, the results of this research are consistent with those of Jinlang Lyu et al. (47). The significant improvements achieved in BMI-Z score, body fat rate, and waist circumference through the comprehensive treatment implemented in the PDCA model in this study provide new empirical support for the conclusion that behavioral therapy combining diet and exercise can significantly reduce central obesity in children. In terms of the treatment implementation scenario, the PDCA model in this study is mainly implemented in medical institutions, which is consistent with the finding in the research of Leonard H. Epstein et al. that the treatment effect in medical institutions is superior to that in schools or communities (48). In terms of indicator selection, this study not only focuses on traditional BMI and waist circumference, but also deeply analyzes body composition, metabolic indicators, and behavioral indicators. This is in line with the conclusion of Clifton J. Holmes et al. on using more comprehensive indicators to evaluate the treatment effect. In terms of treatment methods, the PDCA closed-loop management adopted in this study is similar to structured remote health treatment, although the treatment forms are different, both emphasize the importance of structuring, continuity, and feedback adjustment.

In summary, the PDCA model systematically solves the static and one-way limitations of traditional interventions through the above-mentioned internal mechanisms, thus achieving an effective transformation from behavior change to physiological improvement. Future research can be conducted in the following directions: First, explore the combination of the PDCA model with other effective treatment methods to form multi-dimensional and personalized treatment plans; second, extend the follow-up period to evaluate the long-term maintenance of treatment effects and their true impact on long-term complications; third, deeply analyze the responses of different subgroups to the treatment to provide a basis for precise treatment; fourth, conduct health economics evaluation to assess the cost-effectiveness of the PDCA model; fifth, explore the feasibility and effectiveness of promoting the PDCA model to other chronic disease management fields.

The core advantage of the PDCA closed-loop management model is that it systematically breaks through the three bottlenecks of traditional intervention through a set of dynamic mechanisms. First, it breaks the “Knowledge-action gap” with structured feedback. Its periodic “Check-treatment” link directly links objective indicators such as body fat percentage with behavioral records such as diet diaries, so as to visualize the causal relationship between behaviors and results. This immediate feedback can not only provide positive incentives but also guide doctors and patients to jointly trace and adjust strategies when they fail to meet the standards, so as to internalize external guidance into a driving force for continuous compliance. Secondly, family joint decision-making is used to achieve health empowerment. This model transforms the family role from a passive executor to an active co-decision maker. During the “Planning” phase, goals are jointly set based on the family's reality, and the family participates in recording and reflection throughout the cycle. This process significantly improves the family's self-management ability and sense of responsibility, and it creates a strong, supportive environment for the long-term maintenance of behavior change. Finally, precise interventions are ensured with dynamic adjustments. The dynamic nature of the PDCA cycle allows for flexible adjustments to the protocol based on the results of monthly evaluations. Whether dealing with weight plateaus or actual implementation barriers, this “assess-and-fine-tune” mechanism allows intervention options to be synchronized with the child's real-time progress and the family's specific challenges, allowing for more effective interventions to be implemented, thus, the frustration and loss caused by the rigidity of the plan are avoided, and the continuous improvement of the metabolic index is guaranteed.

## 5 Limitations

This study also has several limitations. First, the retrospective, non-randomized design makes it impossible to avoid selection bias and establish the same strength of causality as a randomized controlled trial. We used strict inclusion and exclusion criteria and presented baseline data that were balanced (see Table 1) to maximize comparability between the two groups Secondly, the study subjects came from a single medical center, and the sample size was relatively limited, which may affect the generalizability of the results. Future studies need to conduct multi-center, large-sample, prospective randomized controlled trials to further verify the effectiveness of the PDCA model. Again, the treatment period was 6 months, although significant improvements were observed, the long-term effects still require longer follow-up assessment. Fourth, some behavioral indicators (such as screen time and snack intake) are dependent on self-report, which may lead to recall bias and social desirability bias. Future research using objective measurement tools such as accelerometers and diet records will make the data more reliable. Fifth, multivariate adjustment for potential confounders (e.g., parental BMI, family socioeconomic status) was lacking in the analysis, which may affect the precision of the effect estimates. Finally, we acknowledge that the more intensive monitoring and attention of the PDCA group itself (e.g., monthly review, wechat group interaction) may produce a “Hawthorne Effect”, whereby study subjects change their behavior because they are aware of being observed. While this is part of structured closed-loop management, it needs to be taken into account when attributing effects. Nevertheless, these limitations do not negate the value of this exploratory research in practice framework construction and preliminary validation, and they point to clear directions for more rigorous research design in the future. Moreover, this study mainly focused on children with primary obesity; for obese children with complex comorbidities, the effectiveness of the PDCA model still needs to be further explored.

## 6 Conclusion

In conclusion, this study integrated the PDCA closed-loop management model into the lifestyle treatment for children with primary obesity, thereby establishing a dynamic, continuous, and individualized health management system. The results showed that this model not only effectively improved the body composition, metabolic parameters, and related behavioral habits of obese children but also achieved significantly better results than conventional management. The PDCA closed-loop management, through its “evaluation—planning—execution—monitoring—feedback—adjustment” cyclic mechanism, successfully broke through the limitations of traditional treatment and provided a valuable practical framework for achieving long-term effective management of childhood obesity. It has important clinical promotion value and public health significance.

## Data Availability

The raw data supporting the conclusions of this article will be made available by the authors, upon request and without undue reservation.
